# Rapid Design of Knowledge-Based Scoring Potentials for Enrichment of Near-Native Geometries in Protein-Protein Docking

**DOI:** 10.1371/journal.pone.0170625

**Published:** 2017-01-24

**Authors:** Alexander Sasse, Sjoerd J. de Vries, Christina E. M. Schindler, Isaure Chauvot de Beauchêne, Martin Zacharias

**Affiliations:** Physik Department T38, Technische Universität München, James-Franck-Straße, Garching, Germany; Friedrich-Alexander-Universitat Erlangen-Nurnberg, GERMANY

## Abstract

Protein-protein docking protocols aim to predict the structures of protein-protein complexes based on the structure of individual partners. Docking protocols usually include several steps of sampling, clustering, refinement and re-scoring. The scoring step is one of the bottlenecks in the performance of many state-of-the-art protocols. The performance of scoring functions depends on the quality of the generated structures and its coupling to the sampling algorithm. A tool kit, GRADSCOPT (GRid Accelerated Directly SCoring OPTimizing), was designed to allow rapid development and optimization of different knowledge-based scoring potentials for specific objectives in protein-protein docking. Different atomistic and coarse-grained potentials can be created by a grid-accelerated directly scoring dependent Monte-Carlo annealing or by a linear regression optimization. We demonstrate that the scoring functions generated by our approach are similar to or even outperform state-of-the-art scoring functions for predicting near-native solutions. Of additional importance, we find that potentials specifically trained to identify the native bound complex perform rather poorly on identifying acceptable or medium quality (near-native) solutions. In contrast, atomistic long-range contact potentials can increase the average fraction of near-native poses by up to a factor 2.5 in the best scored 1% decoys (compared to existing scoring), emphasizing the need of specific docking potentials for different steps in the docking protocol.

## Introduction

Protein interactions play a key role in almost all biological processes [1][2]. While the number of protein-protein interactions discovered by experimental and computational approaches rises rapidly, the number of known complex structures lags behind [3][4]. However, experimental structure determination methods such as nuclear magnetic resonance (NMR) spectroscopy and X-ray crystallography have been used successfully to determine many of the unbound constituents. Protein docking protocols aim to predict the structure of protein complexes from its unbound components. Docking protocols have been developed for single protein-protein, multiple protein, protein-peptide, protein-RNA and protein-DNA interactions [5][6][7][8]. State-of-the-art docking programs often achieve satisfactory results for sampling near-native docking geometries, particularly for cases with no or little structural changes in each constituent during complex formation [9][10].

Docking protocols usually can be divided into two stages: a sampling stage to generate an ensemble of possible complex solutions (decoys) and a scoring stage to select near-native complex structures from the sampled ensemble. However, some docking protocols model conformational adjustments during the binding process by introducing flexibilities during the sampling stage [11][12] and most approaches include a flexible refinement step after the initial rigid body search [13].

The selection of near-native docking solutions usually involves application of one or several combined scoring functions and diverse approaches have been designed to generate successful scoring functions in the last few decades[14][15]. Physical approaches attempt to generate a universally valid scoring function represented by a model for the free energy. A common method is to linearly combine models for each energy contribution. The parameters for these models were fitted to experimentally determined energy values [16][17][18]. However, energy models frequently seem to be insufficient, since they often neglect or oversimplify more complicated terms such as entropic contributions or the change in solvation energy upon binding [19]. Moreover, energy funnels of protein complexes appear to be narrow towards the native structure [20], so that already small deviations on the interface cause large differences in energy. Hence, these methods are less accurate when scoring sampled complex geometries with rather low interface quality.

Knowledge-based scoring functions for protein-protein docking like ITScore-PP [21], Sipper [22], ProBinder [23], DECK [24], DARS [25], Tobi [26] and ATTRACT [8], are based on parameters extracted from the comparison of the scores between near-native or native structures and incorrect poses. One should note that such scoring functions are affected by the structures in the training set and recognize specific structural properties from the sampling algorithm used to generate the decoys [27]. Consequently, docking protocols generally use specific knowledge-based scoring functions for each class of ligands or step in the protocol[6][7][14].

Composite scoring functions such as pyDock [28], RosettaDock [29], HADDOCK [30], Zdock [15][31], FireDock [32], and FiberDock [33] usually use a linear combination of scoring terms to further improve their scoring by accounting for independent complex features. The linear weights for this purpose are determined by optimization or machine learning techniques on a selected set of decoys. In addition, several efforts were made to compare multiple docking protocols and their scoring functions to show future prospects of possible combinations [9].

In general, each of the regarded functions or methods do not seem to work equally well for all cases in the docking benchmark. [10]. Moreover, Vajda and Kozakov pointed out that any scoring function is substantially affected by the properties of the decoys in the training set [27]. Each sampling or refinement method creates a different ensemble of solutions with variant interface characteristics such as different dominant contacts or distances between atom-pairs. As a consequence, scoring functions need to be tailored to the targeted molecules or the sampling algorithm to improve their performance to predict near-native solutions

In this work, we present a tool kit to develop knowledge-based scoring potentials for various decoy sets, sampling methods and problems in protein-docking. The GRid-Accelerated-Directly-SCoring-OPTimizing (GRADSCOPT) tool kit enables a choice between several atomistic and coarse-grained representations for various simple functional forms. The parameters of these potentials can generally be trained on any set of structures of protein complexes by linear regression (LR) models or a directly scoring-dependent Monte-Carlo algorithm (MC). Thanks to pre-calculations of potential-specific feature vectors, re-scoring and re-evaluation of the whole benchmark can be performed very quickly by simple vector multiplications to facilitate the fast testing of several scoring functions and their combinations.

As an application, we designed ten different scoring functions with four different potential forms in our newly defined grouped all atom (GAA) representation. We used unbound rigid-body protein-protein docking decoys obtained from the protein-protein docking program ATTRACT [8]. The performance was evaluated on three different scoring problems on an independent test set consisting of 77 complexes. Furthermore, the contributions of the most distinctive scoring parameters were examined for some of the potentials to explain differences and similarities in their scoring behaviour. Thereby, we found that scoring functions that find a large fraction of near-native solutions favour an increased amount of hydrophobic groups on the interface, especially aromatic rings and end-groups from other hydrophobic side-chains.

We also demonstrate that atomistic potentials trained on near-native solutions from ATTRACT show very different behaviour from potentials that were trained exclusively on the native structure. In addition, we show that potentials using short range interactions for their scoring seem to be more adequate to detect the native bound form whereas potentials using long-range interactions are able to predict on average more near-native solutions in the ensemble of generated decoys.

## Methods

### Set up of scoring benchmark

In this work, we considered 212 protein complexes from the protein-protein docking benchmark 5.0 to form a benchmark for the training of the parameters of our presented scoring potentials [34, 35]. Between 6,000 and 60,000 decoys (depending on the size of the complex) were sampled and scored by ATTRACT's rigid body docking for each complex from its unbound constituents. The scoring benchmark was divided into a training set of 135 complexes and a test set of 77 complexes for evaluation of the genuine performance. We took care that the test and the training sets include approximately the same fractions of hard, medium and rigid body cases to avoid a bias between these two sets in the scoring functions performance (see Tables A and B in S1 File).

### Structural quality assessment

To determine the quality of each structure in the ensemble of sampled solutions, the fraction of native contacts (Fnat), the interface root mean square deviation (Irmsd), the ligand root mean square deviation (Lrmsd) and the CAPRI-stars [35] are calculated for all cases in the benchmark. To assess the quality of protein-protein complexes, we employed the CAPRI quality scheme which was defined by the community wide experiment on the Critical Assessment of PRediction of Interactions (CAPRI). It distinguishes acceptable (*: IRMSD < 4 Å and 0.1 < Fnat< 0.3), medium (**: IRMSD < 2 Å and Fnat > 0.3) and high (***: IRMSD < 1 Å and Fnat > 0.5) quality docking solutions. Generally, acceptable, medium and high quality solutions are summarized under the term near-native solutions to define geometries that are close to the native complex.

### Calculation of potential-specific feature vectors

To ensure fast enumeration of the parameters and also rapid re-scoring afterwards, potential-specific feature vectors are generated in advance for each solution in the decoy sets. The key point is that once a decoy set is given a potential specific feature, e.g. the number of contacts for a given pair of atom types, is fixed and can be pre-calculated. The weight of this feature for the final score can be optimized in a training phase (see below). The content of these feature vectors is generated based on first, the coarse-grained beads or atom types λ assigned to the structures and second, the potential form. All feature vectors contain the sum over potential-specific attributes for each contact-type between two atoms of type A and B. An illustration of the feature vector generation is given in Figure A in S1 File.

The tool kit allows us to generate scoring potentials of different functional forms including contact or step potentials, van-der-Waals (vdw) potentials and potentials that are based on atomistic buried surface areas (BSA). A step potential has a constant value for a contact distance interval between atoms, hence, in this case the feature vectors contain the number of contacts for each decoy in the desired ranges of the steps. For the BSA-potentials the buried surface areas for each coarse-grained or atom type is calculated in advance, by the rolling-probe algorithm using a water radii of 1.4Å [36]. The vectors for the vdw-potentials contain the sum over all distances between two atoms for each contact-type (A,B) to the power of r^-8^ for the repulsive and r^-6^ for the attractive part (see Eq 1).

$$E_{vdw}=\vec{\alpha }\cdot \left(\begin{array}{c} \sum r_{1,1}^{-8}\\ \vdots \\ \sum r_{\lambda ,\lambda }^{-8} \end{array}\right)-\vec{\beta }\cdot \left(\begin{array}{c} \sum r_{1,1}^{-6}\\ \vdots \\ \sum r_{\lambda ,\lambda }^{-6} \end{array}\right);\vec{\alpha }=\vec{\varepsilon }{\vec{\sigma }}^{8},\vec{\beta }=\vec{\varepsilon }{\vec{\sigma }}^{6}$$ Equation 1

The dimension or the number of parameters in the feature vectors is determined by the selected molecular representation. By default, the tool kit offers to use three atomistic representations and the coarse-grained representation used in the ATTRACT docking engine [14]. For the atomistic representations, the atoms of incomplete side-chain residues are rebuilt and atom-types can be assigned according to the optimized potential for liquid simulations (OPLS) [17], according to Tobi et al. [26] or to our newly defined grouped all-atom model (GAA). The GAA representation defines 27 atom types based on the chemical character of each group of amino acids, non-polar, polar, aromatic, positively charged and negatively charged (see Table C in S1 File).

Based on these vectors of each ensemble of solutions, the scoring and re-scoring can be realized by simple and thus quick vector multiplications between the generated potential parameters and the feature vectors of each docking pose (see Eq 1 for vdw potentials). Furthermore, linear regression becomes applicable by fitting the potential parameters to the feature vectors on their quality assessments.

### Parameter training

To train the parameters of the various scoring potentials directly scoring-dependent Monte-Carlo Annealing as well as linear regression models are applicable. The Monte-Carlo Annealing method generates potential parameters by optimizing one of the various default target functions which are based directly on the comparison between the scoring of near-native and false solutions of each complex. For this purpose, quality-weights are assigned to the training decoys, either to distinguish between near-native and incorrect solutions or, depending on the objective, to allocate more influence to higher quality solutions in the training set. As a quality-weight for instance, one can consider the fraction of native contacts (Fnat), the interface root mean square deviation (Irmsd), the ligand root mean square deviation (Lrmsd), the CAPRI-stars [35] (see above) or other alternative assessments, such as DockQ, a recently developed continuous docking quality measure that avoids border effects in its classification scheme [37].

To account for diverse quantities of near-native structures among the complexes, the quality weights are normalized for each complex before optimization. The pre-implemented target functions t are calculated by the sum over the number of all complexes N_c_ and the number of all their training decoys N_d_ of the product between two weights w_r_ and w_q_ that depend on the score and the quality of each decoy i of complex c. The values of the ranking weights can either be set to rise linearly or quadratically from the last position in the decoy set or be assigned after another functional form, depending on the desired objective for the scoring function, such as enriching a fraction of near-natives before refinement or a final ranking.

$$t(\vec{E})=\sum_{c}^{N_{c}}\sum_{i}^{N_{d}}w_{q}^{i,c}\cdot w_{r}(E_{i})$$ Equation 2

In order to perform simulated annealing in parameter space,in each step s the protocol consists of:-changing a randomly picked parameter by a constant or pseudo-temperature T dependent value-re-scoring and re-ranking the decoys for each complex

-calculating a new value for the target-function t_s_(E)

-accepting this step by the temperature dependent probability p^s^_accept_ which is derived from the Metropolis criterion (Eq 3).

$$p_{accept}^{s}=min(1,\frac{e^{t_{s}-t_{s-1}}}{T})$$ Equation 3

After each step, the temperature is decreased by a selected annealing curve down to 0.1% of the starting temperature in a defined number of steps. In this process, the pre-calculated feature vectors make it possible to re-score millions of decoys per second in several steps on a single core.

On the other hand, the linear regression protocol simply fits the parameters on the feature vectors to the negative values or their negative reciprocals of the structural assessments. The resulting potentials assign higher negative values to solutions of higher quality. For that purpose, various regression methods can be considered from the scikit-learn library [38], such as ordinary least squares regression, non-negative least squares regression, support vector regression, robust regressions or Bayesian-Ridge regression. Here, we only show potentials that were generated by ordinary and non-negative least squared regression. Generally, the different linear regressions differ between their cost-functions and some approaches might be more valuable in order to generate simple force-fields on a set of experimentally determined energy values, for instance to predict binding energies or affinities of specific protein complexes [39].

### Performance assessment

For a proper performance evaluation of the generated functions, several simple assessments are performed on an independent test set. We considered the percentage of complexes for which at least one near-native solution can be detected, the average fraction of near-native solutions for each complex and the probability of finding the native structure in the set of generated decoys. The average fraction of near-natives compared to the probability to find one near-native can be used to estimate the specificity of the scoring function towards certain near-native structures in the decoy sets. Finding the native structure within a set of sampled decoys represents an artificial problem and is only used to evaluate the methodology.

### Discriminating scoring contribution

To gain additional insights into the particular scoring performance of BSA-potentials and step-potentials, we regarded the most discriminating parameter contributions dp to the scoring. Analysing the contribution of each parameter allowed us to evaluate differences and similarities between their scoring. As the discriminating parameter contribution for an interaction between atoms of type A and B, we defined the product between the parameter σ_AB_ with the normed absolute difference between the average number of contacts nc (or size of the buried surface area) of near-native (n-nat) and incorrect (incor) solutions.

$$dp_{AB}=\sigma_{AB}\frac{\left|nc_{AB}^{n-nat}-nc_{AB}^{incor}\right|}{nc_{AB}^{tot}}$$ Equation 4

In this regard, one examines the average contribution of each parameter by the distinction between near-native and incorrect solutions, taking into account deviating occurrences of each contact-type in total and between incorrect and near-native solutions. Instead of regarding the absolute difference however, we plotted the contribution p of near-native, native and incorrect solutions separately to detect false positive or false negative contributions, for which the parameter is positive/negative but the average number of contacts is higher in near-native/incorrect solutions. More generally spoken, the sign of these parameters contravenes with the average distribution of contacts between near-native and incorrect solutions and points out a difference between potentials generated by our methodology and statistical potentials.

$$p_{AB}^{nat}=\sigma_{AB}\frac{nc_{AB}^{nat}}{nc_{AB}^{tot}}$$ Equation 5

The discriminating contribution dp of each parameter is given by the difference between the contribution p for incorrect and near-native solutions. The contribution to native complexes is shown as well to see possible differences between the scoring of near-native and native complexes.

### Detailed training recipe for the generation of example scoring potentials

We generated ten potentials in four functional forms: five by linear regression (LR) and five by the Monte Carlo Annealing (MC) method. Two long-range step potentials are based on atom-atom contacts between 0-10Å (MC_gaa_10, LR_gaa_10). Further, two short-range step potentials (MC_gaa_4–6_nat, LR_gaa_4–6_nat) consider two steps between 0-4Å and 4-6Å. The four vdw-like potentials (“_vdw”) use the distance r to the power of r^-8^ for the repulsive and r^-6^ for the attractive parts (soft Lennard-Jones potential), respectively. Since the decoys were generated in a coarse-grained model from ATTRACT, clashes (too close contacts or structural overlap) may result from the change to an atomistic resolution. Hence, for the atomistic vdw-potentials, the contacts between the receptor and the ligand in the range of 0–2 Å were shifted to 2 Å to reduce their influence on the potentials. The potentials based on the buried surface areas consider only heavy atoms (type(“_BSA”)).

All potentials created by the MC approach used an adaptive search for parameter optimization, which altered the parameters by a factor d starting at 1. The temperature was decreased in 300,000 and 100,000 steps respectively from 50 down to 0.05 by a “ziczac” annealing scheme in which the temperature fluctuates between two decreasing exponential functions with a sin²-function. When a convergence criterion was fulfilled in the last 2,000 steps, the search was stopped automatically. For the vdw-potentials the parameter space was restricted. Sigma was kept between 1.5–6 Å and epsilon between 0–50 (see Eq 1). As target-functions for the potentials created by MC, a linearly increasing position weight was used for MC_gaa_4–6_nat, MC_gaa_BSA, MC_gaa_vdw and MC_gaa_vdw_nat. For MC_gaa_10 a quadratically increasing weight was used instead.

For the potentials from the linear regression approach, the fraction of BSAs and contacts was taken as features rather than the absolute number in order to prevent over-fitting on complexes with large interfaces. The potentials LR_gaa_10, LR_gaa_4–6_nat and LR_gaa_BSA were created by an ordinary least squares regression. For the vdw-potentials a non-negative least squares fit was used to generate only positive values for the parameters α = σ⁸ε and β = σ⁶ε.

For all potentials, 5 parameter sets were created by leave-one-out cross-validation. Each parameter set was examined on its validation set to exclude the possibility of potential over-fitting. For step and BSA-potentials, the scaled parameter averages of the five generated sets were taken as our final set. Therefore, each of the five parameter sets was scaled by dividing the parameters through their standard deviation before taking their average. For the vdw-potentials the set of parameters with the highest performance in the validation set was considered to be a consistent choice.

## Results

**Fig 1:**
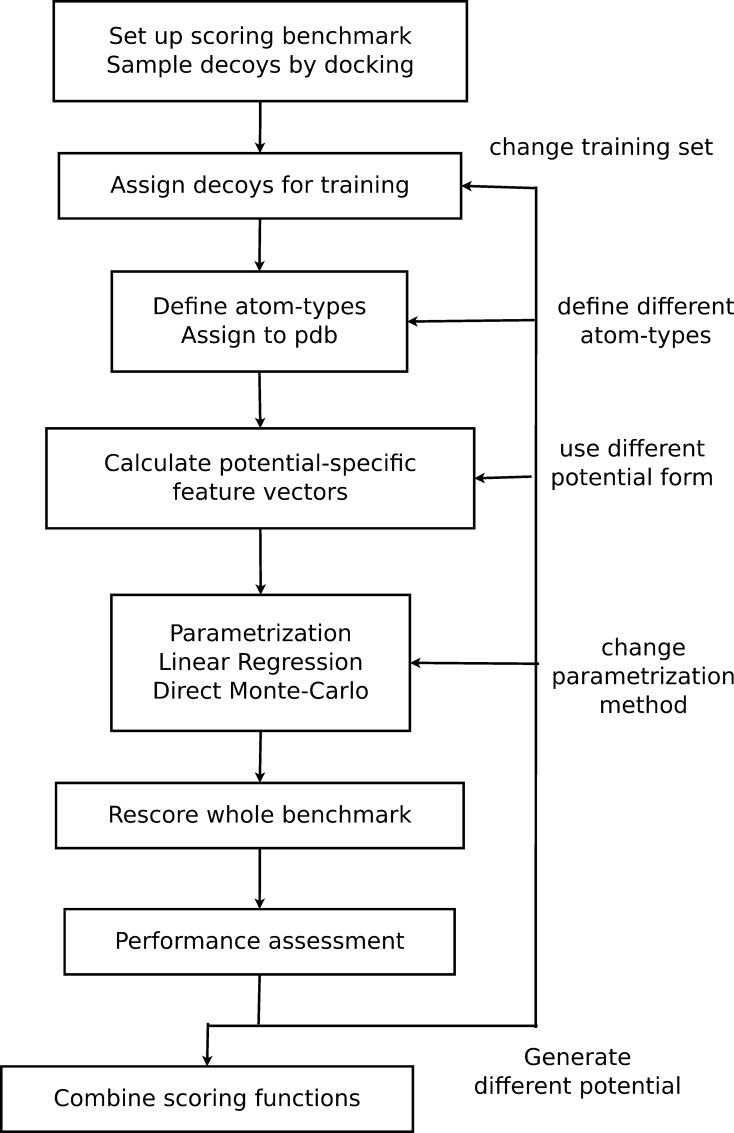
Work-flow to generate a knowledge-based scoring function by the GRADSCOP tool kit. Various scoring potentials can be generated and evaluated in parallel or sequentially by changing the decoy set, the protein representation, the potential form or the training method.

Generating knowledge-based scoring potentials with the GRADSCOPT tool kit involves the following steps (Fig 1). First, a benchmark is set up with a sampling protocol generating an ensemble of decoys for each complex in it. The benchmark is then divided into a training and test set of complexes. Secondly, atom or coarse-grained residue types are assigned to the 3D structures of the receptor and the ligand. With respect to this representation and the form of the desired interaction potential, potential-specific feature vectors are calculated for the generated decoys (see Methods subsection “Calculate potential-specific feature vectors”). Subsequently, the parameters of the potential are trained on a subset of decoys from the training complexes by a directly scoring-dependent Monte-Carlo annealing algorithm or by linear regression. Finally, the whole benchmark is re-scored using the feature vectors of the whole ensemble, and afterwards the scoring performance of the generated potential is evaluated on a training-independent test set. This procedure can be performed in parallel or sequentially to generate several distinct scoring potentials in order to find the best suited variant.

We applied the GRADSCOPT tool kit to design 10 different scoring functions based on three different potential types for unbound rigid protein-protein docking with ATTRACT. All ten scoring potentials are based on the GAA representation of the partners (using the 27 GAA atom types, see Methods and Table C in S1 File). In order to check the dependence of the potentials on the type of optimization method, the parameters were either optimized by the MC or the LR method. (see Table 1, further details on the optimization in Tables D and E in S1 File).

**Table 1:** Overview of the designed potentials, showing the decoy set for training, the parametrization method, the potential form.

Potential	Structures	Method	Functional form
MC_gaa_10	Trained on CAPRI (*,**,***)	MC Position-quadratic	10 A step
MC_gaa_4–6_nat	Trained on native complex	MC Position-linear	4A+ 4–6 A step
MC_gaa_BSA	Trained on CAPRI (*,**,***)	MC Position-linear	BSA-potential
MC_gaa_vdw	Trained on CAPRI (*,**,***)	MC Position-linear	Vdw-potential
MC_gaa_vdw_nat	Trained on native complex	MC Position-linear	Vdw-potential
LR_gaa_10	Trained on CAPRI (*,**,***)	Ordinary least square	10 A step
LR_gaa_4–6_nat	Trained on native structure	Ordinary least square	4A+ 4–6 A step
LR_gaa_BSA	Trained on CAPRI (*,**,***)	Ordinary least square	BSA-potential
LR_gaa_vdw	Trained on CAPRI (*,**,***)	Non-negative least square	Vdw-potential
LR_gaa_vdw_nat	Trained on native structure	Non-negative least square	Vdw-potential

Stars in second column indicate CAPRI quality, see Methods section.

The forms of the presented potentials can be considered as general representatives for the types of scoring functions typically used in the protein-protein docking field: we generated two long-range step potentials with core-core distances between 0-10Å (MC_gaa_10, LR_gaa_10), two short-range step potentials (MC_gaa_4–6_nat, LR_gaa_4–6_nat) with two steps between 0-4Å and 4-6Å, four vdw-like potential (“_vdw”), and two potentials based on the buried surface areas of each atom-type(“_BSA”) (see Table 1 for an overview). To prevent over-fitting, all potentials were created by 5-fold cross-validation and scaled parameter averaging. For the vdw-potentials, the parameter set with the highest performance in the validation set was considered as an appropriate choice. A detailed overview of the parameters used for the generation of the ten scoring potentials is given in Tables D and E in S1 File (see also “Detailed training recipe for the generation of example scoring potentials” in Materials and Methods). The parameter files of the generated potentials are included in the tool kit.

**Fig 2:**
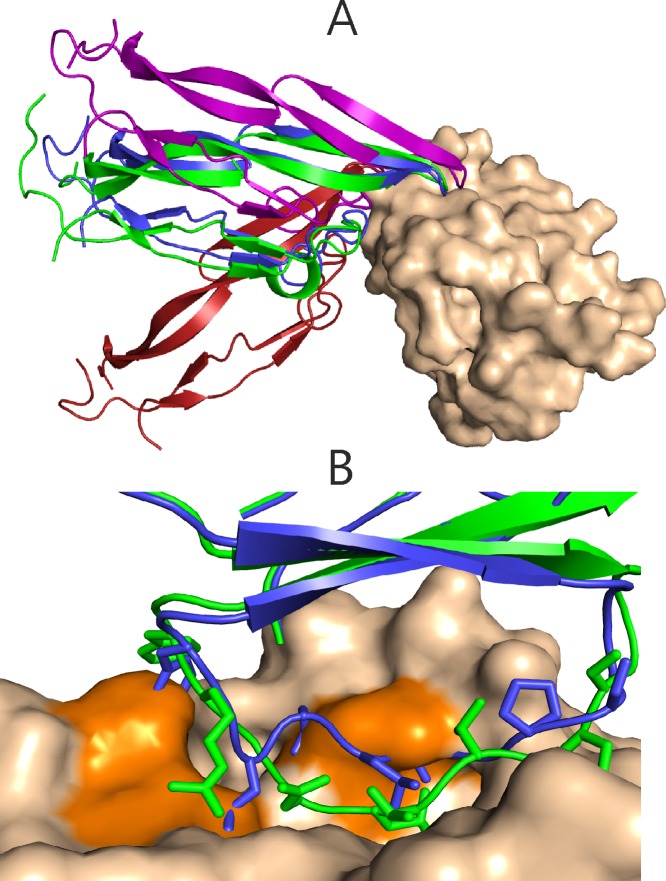
(A) Comparison of the native complex pdb1KTZ (smaller ligand partner protein as green cartoon; receptor protein as beige vdW surface) and examples of acceptable (*)(red cartoon), medium (**) (purple) and high quality (***) (blue) docking solutions (using the CAPRI criteria, see Methods). (B) Clashes (orange) with the unbound chymotrypsin (pdb1ACB, beige vdW-surface) after superposition of the unbound chymotrypsin inhibtor structure (blue) onto the bound form (light green).

In order to investigate the dependence on the structures, defined as correct during training, we trained our potentials to consider all near-native solutions as successes by using the Capri-stars as their quality-weight, or by exclusively giving weights to artificially inserted native structures. The latter potentials were designed only for the case of step potentials (MC/LR_gaa_4–6_nat) and for continuous vdW-potentials (MC/LR_gaa_vdw_nat). It must be pointed out that in a practical docking algorithm, there is a zero probability of sampling the native structure from its unbound constituents and only docking geometries with a significant deviation from the native structure are typically included in the set of solutions. Small deviations between the unbound and bound structure in backbone and side-chain atoms prevent the sampling algorithm from finding the native structure or approaching the native complex structure closely without causing clashes (clashes with the unbound structure at the native position are shown in Fig 2B). Instead, structures with different contacts at the interface are usually formed; this includes non-native contacts and different distances between atoms that are in contact in the native complex. The deviation of acceptable, medium and high quality docking solutions relative to the native structure is illustrated in Fig 2A. Thus, as also emphasized by Vajda et al. [27], to generate a practically useful scoring function for any molecular docking protocol, training should be performed on complex structures sampled from their unbound constituents.

The performances of the generated protein-protein docking scoring potentials were evaluated on a separate test set (77 complexes) by three different assessments: (i) the probability to detect at least one near-native solution; (ii) the probability to detect the inserted native structure; and (iii) the average fraction of near-native geometries in a subset (e.g. enrichment). The performances in these tests were compared to ATTRACT's coarse-grained scoring [14] and to the scoring of Tobi's atomistic short range step potential [26]. As a further control, the performances for near-native structures were also compared to a random scoring.

### Identification of the native structure

**Fig 3:**
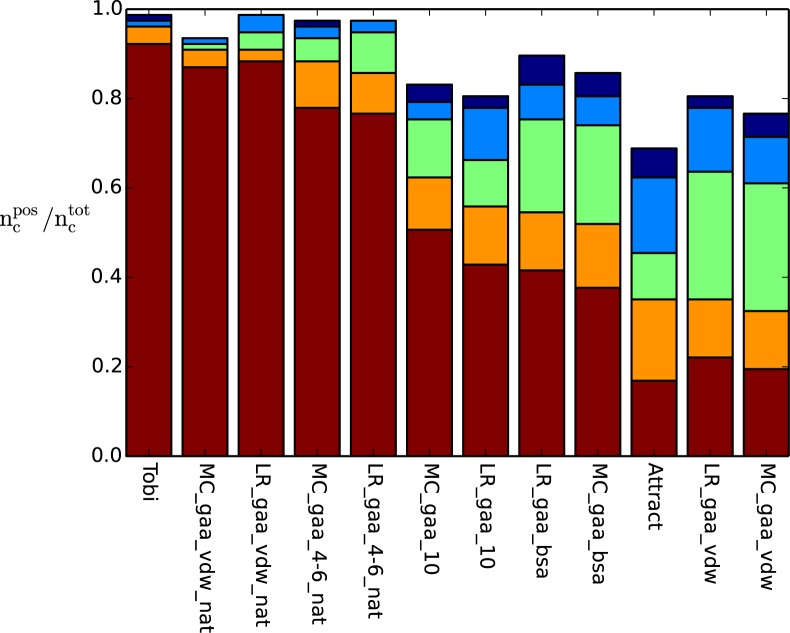
Fraction of test cases (y-axis) for which the native bound complex structure was identified among the decoy complexes in the top 1 (red), top 10 (orange), top 100 (green), top 500 (light blue) and top 1000 (dark blue) using the scoring function indicated at the x-axis. The performances were sorted after the best performing scoring function in the top 10 from left to right.

In order to test the performance of our methodology to generate high quality scoring functions, we first tested the capacity of the designed scoring potentials to identify the native complex structure among all other decoy structures (Fig 3, Tables F and G in S1 File). We expected a scoring function that was trained entirely on native structures could easily solve that problem, since native interface contacts are typically much better aligned than contacts in sampled near-native decoys. Indeed, our potentials trained exclusively to distinguish the native structure yielded very impressive results in predicting the native structure in the top-ranked 10 poses for 88% (two-step potentials) and 91% (vdw-potentials) of the cases (Fig 3). The designed potentials closely approached the performance achieved by the scoring function of Tobi [26]. Other potentials not trained to identify the native structure showed much less impressive results (35–62% top 10 success rate, see Table F and G in S1 File).

### Identification of near-native structures

**Fig 4:**
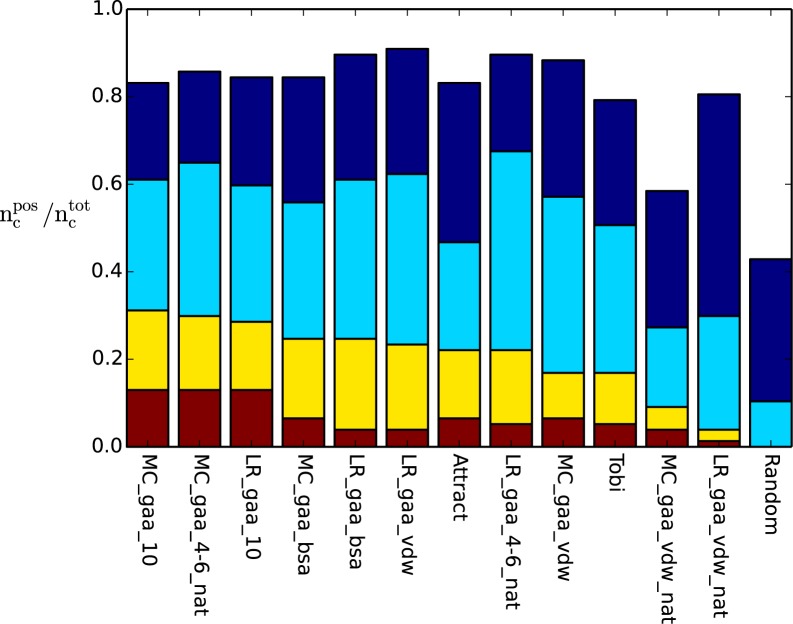
Fraction of test complexes for which at least one near-native (*,**,*** CAPRI-stars) structure was found in the test set in the top 1 (red), top 10 (yellow), top 100 (light blue), top 1000 (dark blue). The performances were sorted after the best performing scoring function in the top 10 from left to right.

In a second evaluation, we tested the ability to identify near-native (that is at least Capri one-star quality) docking solutions among the decoys in the test set (Fig 4, Tables H and I in S1 File). Note, that the decoy set did not include the native complex but only docking solutions obtained from systematic docking using ATTRACT. This corresponds to a realistic protein-protein docking experiment. All optimized potentials scored far better than random. The two 10Å step potentials outperformed ATTRACT by detecting a near-native solution in the top 10 for 31% and 29% of the complexes versus 22% for ATTRACT. The two simple BSA-potentials still predicted a near-native solution for 25% of the cases in the test set. Other functions such as LR_gaa_vdw and MC_gaa_vdw also showed good performances, identifying structures for 62% and 57% of the complexes in the top 100, respectively. Interestingly, the two two-step potentials trained on native structures, MC_gaa_4–6_nat and LR_gaa_4–6_nat, performed very well, predicting a near-native structure in the top 100 for 65% and 68% of the cases. On the other hand, the vdw-potentials that were trained exclusively on the native structure (and showed great performance in identifying the native complex, Fig 3) lacked far behind these results with only 27% and 30% of cases with a near-native solutions scored among the top 100. The comparison between the results for near-native and native structures indicates that vdw-potentials are more sensitive towards certain distances between contacts of the training structures than step potentials. Therefore, the two-step potentials may be able to score near-native solutions well whereas vdw-potentials are unable to show a sufficient scoring. Also here, the results for the training sets were similar to the results in the test set (see Table H and I in S1 File).

### Enrichment of near-native solutions

To improve the quality of docking poses, many groups consider a subsequent refinement of a small fraction of their sampled structures. Refinement protocols allow atoms to adjust to the right position on the interface by introducing more flexibility than in the initial sampling. The chance to generate a high quality structure by refinement increases with the number of near-native structures considered. Therefore, we looked at the average fraction of near-native solutions found in the best scored 0.1%, 1%, 2% and 5% of the decoy sets.

**Fig 5:**
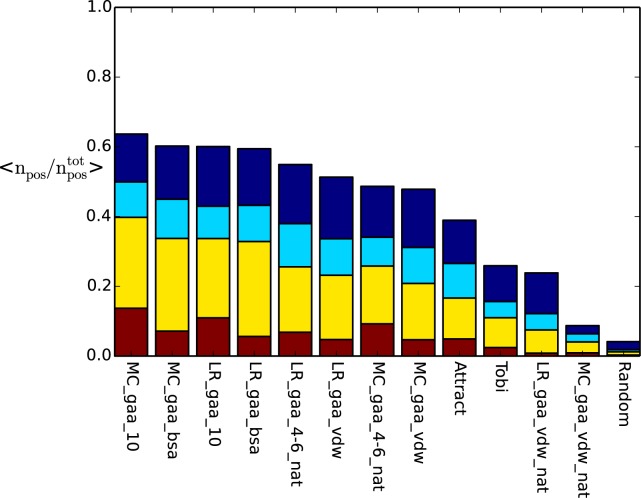
Average fraction of near-native structures (*,**,*** CAPRI-stars) in the decoy set which were identified in the best 0.1 (red), 1 (yellow), 2 (light blue) and 5 (dark blue) % of all decoys in the test set using the scoring functions indicated at the x-axis. The scoring performances were sorted after the fraction of near-native solutions in the top 5% from left to right.

We found that the long-range and the BSA-potentials outperform the standard ATTRACT score by a factor 2.5 and 2 respectively, for the fraction in the best 1% (see Fig 5 and Tables J and K in S1 File). On average they predicted 39% and 33% of all generated near-natives whereas ATTRACT only scores 17% in the top 1%. The vdw-potentials that were trained on near-natives (LR_gaa_vdw and MC_gaa_vdw) predicted slightly more near-natives as ATTRACT with about 50% in the top 5% compared to 39%(out of 6,000 to 60,000 decoys). Thus, MC_gaa_10 predicts on average as many near-natives in the top 1% as ATTRACT in the top 5%. Again, most potentials trained to find only the native structure performed worse: The potential by Tobi et al. and the vdw-potentials identified on average less than 26% of the near-native structures in the best scoring 5%, while the two-step potentials still placed 48% and 54% of the near-natives in the top 5%.

Comparing the long-range step and the BSA-potentials, we observed that potentials created by Monte-Carlo Annealing worked slightly better than their counterpart from ordinary least squares fit. This might result from the target function in the MC algorithm that aims at improving the score of all near-native structures in the decoy set. The results further indicate that compared to atomistic or coarse-grained vdw-potentials, simple long-range step potentials and BSA-potentials are more likely to identify structurally diverse near-native complexes. Both our created atomistic and the coarse-grained vdw-potential from ATTRACT seem to be more specific towards structures of higher quality since contacts on the interface have to be placed at a certain distance in order to achieve a good score. Hence, vdw-potentials generally do not seem to be a good choice to deal with the diversity of the different near-native solutions of each complex.

### Discriminating parameter contributions to near-native enrichment for BSA- and long-range step potentials

After the designed long-range step potentials and the BSA-potentials achievee impressive results for the fraction of near-native solutions, we wanted to investigate their scoring in more detail. We analysed the 20 most positive and negative discriminating scoring contributions (dp) for the step potentials along with all the scoring contributions of the BSA-potentials (see Discriminating scoring contribution). We looked at the difference between the average contribution p of each parameter of near-native (green) and incorrect solutions (red) to estimate its distinctive power in the scoring. By multiplying the parameters with their normed features, we accounted for the deviating occurrences of each contact-type in general and between incorrect and near-native solutions. First, we considered the discriminating scoring contributions for the 23 parameters of the BSA-potentials, MC_gaa_BSA (Figure B in S1 File) and LR_gaa_BSA (Figure B in S1 File). We found both potentials mainly predict near-native structures on increased BSAs of (I) C-rings and CH2 groups from aromatic side chains, (ii) CH3, CH2, CH, and S groups from nonpolar residues and (iii) nitrogen from positively charged amino acids as well as on reduced BSAs of CH2 groups from negatively and positively charged amino acids. The two potentials seem to deviate only slightly in the strength of contributions from less discriminating atom-types, for which the near-native and incorrect contributions are almost equal.

**Fig 6:**
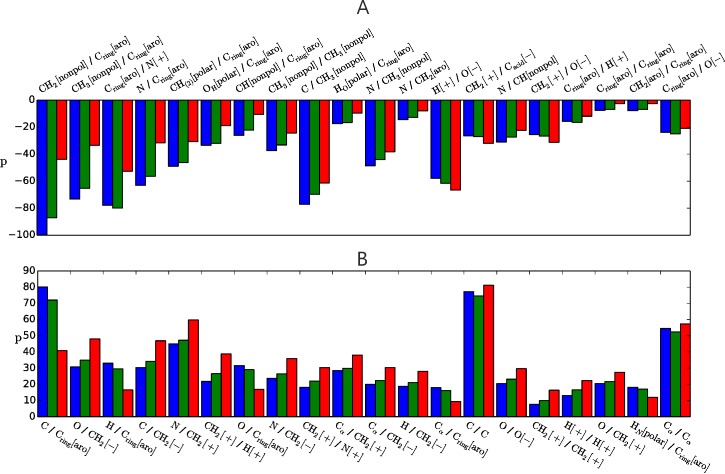
20 most negative (A) and most positive (B) scoring contributions of the parameter (x-axis) for the MC_gaa_10 potential shown for the average native (blue), near-native (green) and incorrect (red) solution. The discriminating scoring contribution is defined as described in the methods section by the difference between the near-native and incorrect contributions (red and green).

When we looked at the most negative discriminating parameter contributions of the 10Å step potentials, MC_gaa_10 and LR_gaa_10 (Fig 6A, Figure C in S1 File), we detected similarities to the BSA-potentials as well as between the two potentials themselves. Eight and seven contributions respectively were between atom-types that were also most significant for the BSA-potentials. Seventeen and fifteen out of the 20 most favourable contributions involved at least one group from a hydrophobic residue. The two potentials still showed major differences in the 20 most positive parameter contributions (Fig 6B, Figure C in S1 File): The MC_gaa_10 potential included eleven contributions which involved at least one CH2 group of charged amino acids. Their large positive contributions represent a penalty for charged residues to appear at the interface.The LR_gaa_10 potential only includes two of those positive contributions. Additionally, the LR_gaa_10 potential indicates eleven strongly false positive contributions whereas MC_gaa_10 shows only four. False negative/positive contributions were defined as contributions with a negative/positive parameter value but their average number of contacts is higher in incorrect/near-native structures. However, when we changed the signs of parameters with false-positive or false-negative contributions, the overall scoring performance to enrich a subset with near-native structures got worse (data not shown), indicating a difference of our potentials to statistically derived potentials. In comparing the average scoring contribution of each parameter between native complexes (blue), incorrect solutions and near-native solutions, we found scoring contributions to native and near-native structures to be very similar. Nevertheless, the discriminating scoring contribution dp to incorrect solutions is generally larger to native structures, indicating one reason for the better scoring performance of potentials for native structures than near-natives.

## Discussion

The performance of the generated scoring potentials for protein-protein docking showed that both our approaches were able to rapidly create high quality scoring potentials. All our potentials worked significantly better than a random scoring; they even outperformed or competed with two state-of-the-art functions in all three presented assessments. The continuous vdw-potentials performed extremely well in scoring the native structure but poorly for enriching near-native docking solutions. Very similar results were found for the popular Tobi score. These results supported our idea that vdw-potentials are extremely dependant on the distances of interface atoms in their training structures and hence are biased towards certain near-natives or natives in the decoy set.

The scoring performances of our generated potentials for at least one near-native solution are in general comparable to the results obtained from ZDOCK[40], ClusPro and SwarmDock [41]. For example, our generated potential MC_gaa_10 placed a near-native structure for 13% of the cases at rank 1, for 31% in the top 10 and for 61% in the top 100 compared to 10%, 36% and 65% for SwarmDock.

Furthermore, we showed that long-range step potentials and simple potentials based on the atomistic buried surface areas were able to detect on average up to 2.5 times more near-native structures in the top 1% than the standard ATTRACT score. Therefore, these potentials seem to be well applicable for selecting a subset of structures for a subsequent refinement in the ATTRACT or other docking protocols. No significant improvement upon combining these potentials was found (not shown) indicating that similar characteristics are evaluated.

Interestingly, the analysis of the parameter contributions for these two types of potentials indicated that not only specific contacts between groups from aromatic and non-polar residues were favoured by the step potentials but also contacts between these groups with polar and charged residues or backbone atoms. The preference of these contacts by long-range step potentials may account for the increased presence of these groups in protein-protein interfaces in general as also detected by the BSA-potentials. This seems to be helpful to select many near-natives since interfaces of near-natives with lower quality may not be aligned well enough to select them exclusively on chemically specific contacts.

As a result, we find that it would be beneficial to use several scoring functions before and after a refinement to, first enrich near-native solutions and, secondly to predict a high quality solution. Based on our results, we suggest that unspecific long-range step or BSA-potentials should be applied for an enrichment of near-native structures after sampling. On the other hand, short-range or atomistic-distance dependent potentials, such as our vdw-potentials and our multiple step potentials, performed very well for native structures. Thus, applying first long-range potentials to select structures for refinement and afterwards potentials, trained to recognize the highest quality solutions as a final scoring, may further improve the quality of the structures and also the total scoring performance of the protocol.

Finally, our results show that both of our methods are robust and offer viable strategies to generate problem-adapted solutions, as they are needed in practical docking problems. Due to the usage of pre-calculated feature vectors for each potential form, the tool kit enables the user to generate and evaluate various scoring functions rapidly to find a problem-adapted solution. Moreover, it also allows the user to optimize the functional form of the scoring potential (e.g. with respect to inter-atomic distances), as we found that the functional form that performs best depends very strongly on the scoring problem. Other methods to create scoring potentials, such as linear programming [42][43] possess the advantage of numerically generating a globally optimal set of parameters with respect to the defined target function. However, the handling of these approaches appears to be more complex and their applicability seems to be narrower since it requires a set of training structures that do not impose impossible conditions on its constraints [26]. Especially for the training on near-native solutions with diverse structures and interface compositions or larger benchmarks with diverse types of protein-interactions, finding a set of restricting constraints can become difficult or impossible without excluding some important decoys.

The tool kit, a detailed manual with example executive scripts, the ATTRACT rigid-body protein-protein docking benchmark and parameter files for the created potentials can be found at http://www.t38.ph.tum.de/-> Downloads.

## Supporting Information

S1 File **Figure A** Schematic illustration of the pre-calculated grids for each protein complex in the benchmark containing potential-specific feature vectors. The score E of each decoy can be obtained by a vector multiplication with the potential parameters. All feature vectors are stored in a grid for all decoys of each complex and are used to accelerate re-scoring during training and assessment.
**Figure B** The 23 scoring contributions of the parameter (x-axis) to the MC_gaa_BSA (A) or LR_gaa_BSA (B) scoring potential shown for the average native (blue), near-native (green) and incorrect (red) solution. The discriminating scoring contribution is defined as described in the methods section by the difference between the near-native and incorrect contributions (red and green).
**Figure C** 20 most negative (A) and most positive (B) scoring contributions of the parameter (x-axis) for the LR_gaa_10 potential shown for the average native (blue), near-native (green) and incorrect (red) solution. The discriminating scoring contribution is defined as described in the methods section by the difference between the near-native and incorrect contributions (red and green).
**Tables A Protein data bank entries for training set of protein-protein complexes.** Protein databank (pdb) entries of the training set consisting of 135 protein-protein complexes used for the parameter generation.
**Table B Protein data bank entries for test set of protein-protein complexes.** Protein databank (pdb) entries of the test set consisting of 77 protein-protein complexes.
**Table C List of atom-types in the grouped-all atom (GAA) representation.** Assignment of the 27 atom types of the GAA representation.
**Tables D Parameters for scoring potential generation using Monte Carlo Simulated Annealing**.
**Table E Parameters for scoring potential generation using linear regression**.
**Tables F Performance of designed scoring potentials for identification of native docking solutions in the training set**.
**Table G Performance of designed scoring potentials for identification of native docking solutions in the test set**.
**Tables H Performance of designed scoring potentials for identification of near-native docking solutions in the training set**.
**Table I Performance of designed scoring potentials for identification of near-native docking solutions in the test set**.
**Tables J Ranking of near-native docking solutions by different scoring functions in the training set**.
**Table K Ranking of near-native docking solutions by different scoring functions in the test set**.
(DOCX)
