# Supplementary material for: Rapid Design of Knowledge-Based Scoring Potentials for Enrichment of Near-Native Geometries in Protein-Protein Docking
Source: PLoS One. 2017 Jan 24;12(1):e0170625. doi: 10.1371/journal.pone.0170625 (PMC5261736; doi:10.1371/journal.pone.0170625)
Supplement: S1 File — Figure A Schematic illustration of the pre-calculated grids for each protein complex in the benchmark containing potential-specific feature vectors. The score E of each decoy can be obtained by a vector multiplication with the potential parameters. All feature vectors are stored in a grid for all decoys of each complex and are used to accelerate re-scoring during training and assessment. Figure B The 23 scoring contributions of the parameter (x-axis) to the MC_gaa_BSA (A) or LR_gaa_BSA (B) scoring potential shown for the average native (blue), near-native (green) and incorrect (red) solution. The discriminating scoring contribution is defined as described in the methods section by the difference between the near-native and incorrect contributions (red and green). Figure C 20 most negative (A) and most positive (B) scoring contributions of the parameter (x-axis) for the LR_gaa_10 potential shown for the average native (blue), near-native (green) and incorrect (red) solution. The discriminating scoring contribution is defined as described in the methods section by the difference between the near-native and incorrect contributions (red and green). Tables A Protein data bank entries for training set of protein-protein complexes. Protein databank (pdb) entries of the training set consisting of 135 protein-protein complexes used for the parameter generation. Table B Protein data bank entries for test set of protein-protein complexes. Protein databank (pdb) entries of the test set consisting of 77 protein-protein complexes. Table C List of atom-types in the grouped-all atom (GAA) representation. Assignment of the 27 atom types of the GAA representation. Tables D Parameters for scoring potential generation using Monte Carlo Simulated Annealing. Table E Parameters for scoring potential generation using linear regression. Tables F Performance of designed scoring potentials for identification of native docking solutions in the training set. Table G Performance of designed scor [file pone.0170625.s001.docx]

Supporting Information:

Rapid design of knowledge-based scoring potentials for enrichment of near-native geometries in protein docking

Alexander Sasse, Sjoerd J. de Vries, Christina E.M. Schindler, Isaure Chauvot de Beauchêne & Martin Zacharias*

Physik Department T38, Technische Universität München, James-Franck-Straße 1, 85748 Garching, Germany

*) corresponding author:

Martin Zacharias

Physik Department T38

Technische Universität München

James-Franck-Straße 1

85748 Garching

e-mail: [zacharias@tum.de](mailto:zacharias@tum.de#_blank)


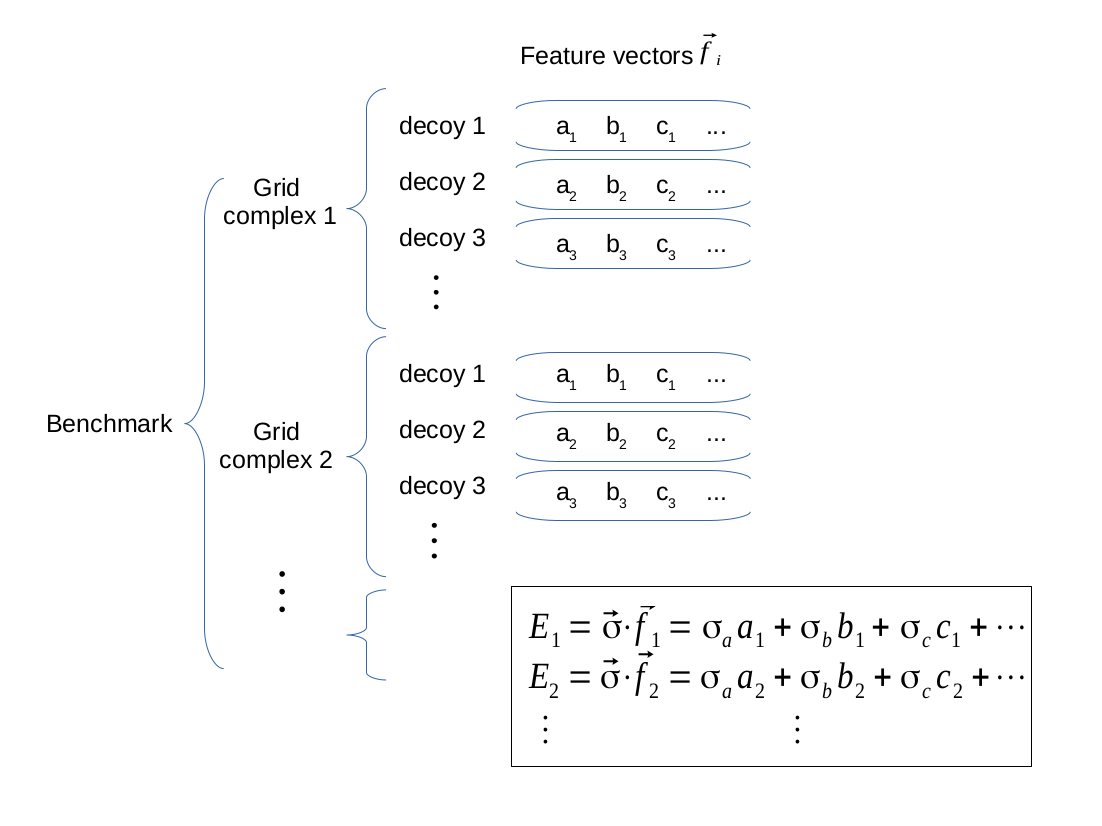


**Figure A.** Schematic illustration of the pre-calculated grids for each protein complex in the benchmark containing potential-specific feature vectors. The score E of each decoy can be obtained by a vector multiplication with the potential parameters. All feature vectors are stored in a grid for all decoys of each complex and are used to accelerate re-scoring during training and assessment.

(A)

(B)

**Figure B.** (A) The 23 scoring contributions of the parameter (x-axis) to the MC_gaa_BSA scoring potential (B) same LR_gaa_BSA potential shown for the average native (blue), near-native (green) and incorrect (red) solution. The discriminating scoring contribution is defined as described in the methods section by the difference between the near-native and incorrect contributions (red and green)

(A)

(B)

**Figure C.** (A) 20 most negative scoring contributions of the parameter (x-axis) and (B) 20 most positive scoring contributions for the LR_gaa_10 potential shown for the average native (blue), near-native (green) and incorrect (red) solution. The discriminating scoring contribution is defined as described in the methods section by the difference between the near-native and incorrect contributions (red and green).

**Table A.** Protein databank (pdb) entries of the training set consisting of 135 protein-protein complexes used for the parameter generation and the assigned difficulty according to the protein docking benchmark 5.0

**hard (15):** 1E4K, 2HMI, 1FQ1, 1PXV, 1ZLI, 2O3B, 1ATN, 1IBR, 1JK9, 1JMO ,1JZD, 2I9B, 2IDO, 2OT3, 1ACB

**medium (22):** 1IJK, 1JIW, 1KKL, 1M10, 1GP2, 1GRN, 1HE8, 1IB1, 1K5D, 1LFD, 1MQ8, 1R6Q, 1SYX, 1WQ1, 1XQS, 2CFH, 2J7P, 2OZA, 2Z0E, 1ZM4, 1CGI, 1FC2

**rigid (98):** 1AHW, 1BVK, 1DQJ, 1E6J, 1JPS, 1MLC, 1VFB, 1WEJ, 2FD6, 2I25, 1I9R, 1IQD, 1K4C, 1KXQ, 1NCA, 1NSN, 2JEL, 1AVX, 1AY7, 1BVN, 1CLV, 1D6R, 1DFJ, 1E6E, 1EAW, 1EWY, 1EZU, 1F34, 1FLE, 1GL1, 1GXD, 1HIA, 1JTG, 1MAH, 1N8O, 1OC0, 1OPH, 1PPE, 1R0R, 2B42, 2J0T, 2MTA, 2O8V, 2OUL, 2PCC, 2SIC, 2SNI, 2UUY, 3SGQ, 7CEI, 1A2K, 1AK4, 1AKJ, 1AZS, 1EFN, 1F51, 1FCC, 1FFW, 1FQJ, 1GCQ, 1GLA, 1GPW, 1H9D, 1HCF, 1HE1, 1I4D, 1J2J, 1ML0, 1OFU, 1OYV, 1PVH, 1QA9, 1QFW, 1RLB, 1RV6, 1S1Q, 1SBB, 1T6B, 1US7, 1WDW, 1XD3, 1XU1, 1Z0K, 2A9K, 2AJF, 2B4J, 2BTF, 2FJU, 2G77, 2HLE, 2HQS, 2OOB, 2OOR, 2VDB, 3BP8, 3D5S, 2AYO, 4CPA

**Table B.** Pdb entries of 77 protein-protein complexes that formed the test set for the assessment of the generated potentials and the assigned difficulty according to the protein docking benchmark 5.0

**hard (8):** 2C0L, 1EER, 1BKD, 3H11, 1RKE, 3L89, 3F1P, 3FN1

**medium (22):** 1I2M, 3CPH, 2H7V, 1NW9, 2HRK, 3DAW, 3G6D, 3SZK, 4FZA, 4IZ7, 3AAA, 3EO1, 3L5W, 3V6Z, 4JCV, 3AAD, 3R9A, 4LW4, 3HI6, 3BX7, 3S9D, 1B6C

**rigid (47):** 2VIS, 1BJ1, 1FSK, 1TMQ, 1UDI, 1YVB, 2ABZ, 1GHQ, 1JWH, 1K74, 1KAC, 1KLU, 1KTZ, 1KXP, 1Z5Y, 1ZHH, 1ZHI, 2A5T, 1BUH, 1E96, 1JTD, 2GTP, 3A4S, 3K75, 3P57, 1M27, 2VXT, 3PC8, 4G6J, 2W9E, 3EOA, 3H2V, 3VLB, 4G6M, 2A1A, 2X9A, 3BIW, 3LVK, 3RVW, 4DN4, 4H03, 4M76, 2GAF, 2YVJ, 3HMX, 3MXW, 4HX3

**Table C.** List of atom-types in the grouped-all atom representation: The atoms are divided into 5 groups of amino acids, nonpolar, polar, aromatic, positively charged (+) and negatively charged (-), and among these groups of residues atom-types are assigned to similar groups.

| Atom-type | Residue | Group |
| --- | --- | --- |
| 1 | Backbone | N |
| 2 | Backbone | H |
| 3 | Backbone | CA |
| 4 | Backbone | C |
| 5 | Backbone | O |
| 6 | Ala, Val, Leu, Ile, Met, Thr | CH_3_[nonpolar] |
| 7 | Pro, Leu, Ile, Met | CH_2_[nonpolar] |
| 8 | Val, Leu | CH[nonpolar] |
| 9 | Met | S[nonpolar ] |
| 10 | Ser, Cys, Asp, Gln | CH_(2)_[polar] |
| 11 | Ser, Thr, Tyr | O(OH)[polar] |
| 12 | Ser, Thr, Cys, Tyr | H(OH)[polar] |
| 13 | Cys | S[polar] |
| 14 | Asp, Gln | O=[polar] |
| 15 | Asp, Gln | N(NH_(2)_)[polar] |
| 16 | Asp, Gln | H(NH_(2)_)[polar] |
| 17 | Asp, Gln | C=[polar] |
| 18 | Phe, Tyr, Trp | CH_(2)_[aromatic ] |
| 19 | Phe, Tyr, Trp | Cπ[aro matic] |
| 20 | Arg, His | C(H)[+] |
| 21 | Lys, Arg, His | CH_2_[+] |
| 22 | Lys, Arg, His | N[+] |
| 23 | Lys, Arg, His | H[+] |
| 24 | Asp, Glu | O(COOH)[-] |
| 25 | Asp, Glu | CH_2_[-] |
| 26 | Asp, Glu | C(COOH)[-] |
| 27 | Gly | C[Glycine] |

**Table D.** Parameters to generate potentials by directly scoring dependent Monte-Carlo Annealing

|  | Decoy set | Quality weights | MC annealing | Target-function | Search space | Representation | Potential | choice |
| --- | --- | --- | --- | --- | --- | --- | --- | --- |
| MC_gaa_10 | 3000 best scored + all near-native (1-, 2-, 3-CAPRI stars) Attract unbound rigid | 1, 2, 3 CAPRI-stars | Ziczac T0=100, 100.000 steps, convergence after 1000 | Position-quadratic | Adaptive from 1.0 | Atomistic 27 atom types form GAA | 10 A step | Average, normalized by standard deviation |
| MC_gaa_4-6_nat | 3000 best scored + all near native Attract unbound rigid + native | Native complex | Ziczac T0=30, 100.000 steps, convergence after 1000 | Position-linear | Adaptive from 1.0 | Atomistic 27 atom types form GAA | 4A and 4 to 6 A step | Average, normalized by standard deviation |
| MC_gaa_BSA | 3000 best scored + all near native Attract unbound rigid | 1, 2, 3 CAPRI-stars | Ziczac T0=20, 100.000 steps, convergence after 2000 | Position-linear | Adaptive from 0.5 | Atomistic 27 atom types form GAA | Buried Surface Area | Average, normalized by standard deviation |
| MC_gaa_vdw | 3000 best scored + all near native Attract unbound rigid | 1, 2, 3 CAPRI-stars | Ziczac T0=50, 300.000 steps, convergence after 3000 | Position-linear | Adaptive from 1.0 constraints: sigma = [1.5, 6.], epsilon = [0, 50] | Atomistic 27 atom types form GAA | Lennard-Jones-like to power -8 and -6 | Cross-validation set 3* |
| MC_gaa_vdw_nat | 3000 best scored + all near native Attract unbound rigid +native | Native complex | Ziczac T0=50, 300.000 steps, convergence after 3000 | Position-linear | Adaptive from 1.0 constraints: sigma = [1.5, 3.5], epsilon = [0, 50] | Atomistic 27 atom types form GAA | Lennard-Jones-like to power -8 and -6 | Cross-validation set 2* |

**Table E.** Parameters to generate potentials by Linear Regression

|  | Decoy set | Target values | Regression | Representation | Potential | Choice |
| --- | --- | --- | --- | --- | --- | --- |
| LR_gaa_10 | 3000 best scored + all near native Attract unbound rigid | 1, 2, 3 CAPRI-stars negative | Ordinary least square | Atomistic 27 atom types form GAA | 10 A step, normalized by total mean of complex | Average, normalized by standard deviation |
| LR_gaa_4-6_nat | 3000 best scored + all near native Attract unbound rigid + native | Native structure as -4 | Ordinary least square | Atomistic 27 atom types form GAA | 4A and 4 to 6 A step, normalized by total mean of complex | Average, normalized by standard deviation |
| LR_gaa_BSA | 3000 best scored + all near native Attract unbound rigid | 1, 2, 3 CAPRI-stars negative | Ordinary least square | Atomistic 27 atom types form GAA | Buried Surface Area, normalized by total mean of complex | Average, normalized by standard deviation |
| LR_gaa_vdw | 3000 best scored + all near native Attract unbound rigid | 1, 2, 3 CAPRI-stars negative | Non-negative least square | Atomistic 27 atom types form GAA | Lennard-Jones-like to power -8 and -6 | Cross-validation set 2* |
| LR_gaa_vdw_nat | 3000 best scored + all near native Attract unbound rigid + native | Native structure as -4 | Non-negative least square | Atomistic 27 atom types form GAA | Lennard-Jones-like to power -8 and -6 | Cross-validation set 2* |

*: Cross-validation sets consisted out of 4/5 of complexes in the training set. The training set was divided into 5 equally long subsets of complexes and one subset was excluded for each cross-validation set. The following subsets were excluded for the cross-validation sets 1-5:

**1:** 1WDW, 1XD3, 1XU1, 1Z0K, 2A9K, 2AJF, 2B4J, 2BTF, 2FJU, 2G77, 2HLE, 2HQS, 2OOB, 2OOR, 2VDB, 3BP8, 3D5S, 2AYO, 4CPA, 1ACB, 1IJK, 1JIW, 1KKL, 1M10, 1GP2, 1GRN, 1HE8

**2:** 1AZS, 1EFN, 1F51, 1FC2, 1FCC, 1FFW, 1FQJ, 1GCQ, 1GLA, 1GPW, 1H9D, 1HCF, 1HE1, 1I4D, 1J2J, 1ML0, 1OFU, 1OYV, 1PVH, 1QA9, 1QFW, 1RLB, 1RV6, 1S1Q, 1SBB, 1T6B, 1US7

**3:** 1EZU, 1F34, 1FLE, 1GL1, 1GXD, 1HIA, 1JTG, 1MAH, 1N8O, 1OC0, 1OPH, 1PPE, 1R0R, 2B42, 2J0T, 2MTA, 2O8V, 2OUL, 2PCC, 2SIC, 2SNI, 2UUY, 3SGQ, 7CEI, 1A2K, 1AK4, 1AKJ

**4:** 1AHW, 1BVK, 1DQJ, 1E6J, 1JPS, 1MLC, 1VFB, 1WEJ, 2FD6, 2I25, 1I9R, 1IQD, 1K4C, 1KXQ, 1NCA, 1NSN, 2JEL, 1AVX, 1AY7, 1BVN, 1CGI, 1CLV, 1D6R, 1DFJ, 1E6E, 1EAW, 1EWY

**5:** 1IB1, 1K5D, 1LFD, 1MQ8, 1R6Q, 1SYX, 1WQ1, 1XQS, 2CFH, 2J7P, 2OZA, 2Z0E, 1ZM4, 1E4K, 2HMI, 1FQ1, 1PXV, 1ZLI, 2O3B, 1ATN, 1IBR, 1JK9, 1JMO, 1JZD, 2I9B, 2IDO, 2OT3

**Table F.** Percentage of 135 complexes in the training set for which the artificially inserted native structure can be found in the top 1, top 10, top 100, top 200, top 500, top 1000, and top 2000.

|  | top 1 | 10 | 100 | 200 | 500 | 1000 | 2000 |
| --- | --- | --- | --- | --- | --- | --- | --- |
| Attract | 24.44 | 41.48 | 63.7 | 69.63 | 78.52 | 85.93 | 88.15 |
| Tobi | 94.81 | 97.04 | 97.04 | 97.78 | 98.52 | 98.52 | 98.52 |
| MC_gaa_10 | 42.22 | 58.52 | 68.89 | 76.3 | 82.96 | 87.41 | 89.63 |
| LR_gaa_10 | 49.63 | 65.19 | 78.52 | 80.0 | 86.67 | 89.63 | 94.07 |
| MC_gaa_BSA | 40.0 | 51.11 | 63.7 | 68.89 | 76.3 | 82.22 | 85.19 |
| LR_gaa_BSA | 42.22 | 54.07 | 68.15 | 75.56 | 81.48 | 85.93 | 90.37 |
| MC_gaa_vdw | 20.74 | 33.33 | 54.81 | 63.7 | 71.11 | 79.26 | 82.96 |
| LR_gaa_vdw | 27.41 | 41.48 | 62.96 | 71.11 | 78.52 | 85.19 | 88.89 |
| MC_gaa_4-6_nat | 94.81 | 99.26 | 100.0 | 100.0 | 100.0 | 100.0 | 100.0 |
| LR_gaa_4-6_nat | 80.0 | 91.85 | 97.78 | 99.26 | 99.26 | 100.0 | 100.0 |
| MC_gaa_vdw_nat | 83.7 | 86.67 | 94.81 | 95.56 | 96.3 | 97.78 | 98.52 |
| LR_gaa_vdw_nat | 97.04 | 97.78 | 99.26 | 99.26 | 99.26 | 99.26 | 100.0 |

**Table G.** Percentage of 77 complexes in the test set for which the artificially inserted native structure can be found in the top 1, top 10, top 100, top 200, top 500, top 1000, and top 2000.

|  | top 1 | 10 | 100 | 200 | 500 | 1000 | 2000 |
| --- | --- | --- | --- | --- | --- | --- | --- |
| Attract | 16.88 | 35.06 | 45.45 | 54.55 | 62.34 | 68.83 | 76.62 |
| Tobi | 92.21 | 96.1 | 96.1 | 96.1 | 97.4 | 98.7 | 98.7 |
| MC_gaa_10 | 50.65 | 62.34 | 75.32 | 77.92 | 79.22 | 83.12 | 84.42 |
| LR_gaa_10 | 42.86 | 55.84 | 66.23 | 72.73 | 77.92 | 80.52 | 84.42 |
| MC_gaa_BSA | 37.66 | 51.95 | 74.03 | 77.92 | 80.52 | 85.71 | 88.31 |
| LR_gaa_BSA | 41.56 | 54.55 | 75.32 | 79.22 | 83.12 | 89.61 | 92.21 |
| MC_gaa_vdw | 19.48 | 32.47 | 61.04 | 67.53 | 71.43 | 76.62 | 80.52 |
| LR_gaa_vdw | 22.08 | 35.06 | 63.64 | 70.13 | 77.92 | 80.52 | 83.12 |
| MC_gaa_4-6_nat | 77.92 | 88.31 | 93.51 | 94.81 | 96.1 | 97.4 | 97.4 |
| LR_gaa_4-6_nat | 76.62 | 85.71 | 94.81 | 97.4 | 97.4 | 97.4 | 97.4 |
| MC_gaa_vdw_nat | 87.01 | 90.91 | 92.21 | 92.21 | 93.51 | 93.51 | 94.81 |
| LR_gaa_vdw_nat | 88.31 | 90.91 | 94.81 | 98.7 | 98.7 | 98.7 | 98.7 |

**Table H.** Percentage of 135 complexes in the training set for which at least one near-native solution can be found in the top 1, top 10, top 100, top 200, top 500, top 1000, and top 2000.

|  | top 1 | 10 | 100 | 200 | 500 | 1000 | 2000 |
| --- | --- | --- | --- | --- | --- | --- | --- |
| Random | 0.0 | 2.22 | 19.26 | 31.11 | 48.89 | 71.11 | 85.93 |
| Attract | 8.15 | 28.15 | 64.44 | 74.81 | 88.15 | 92.59 | 95.56 |
| Tobi | 4.44 | 24.44 | 56.3 | 69.63 | 80.74 | 88.15 | 92.59 |
| MC_gaa_10 | 15.56 | 34.07 | 65.93 | 74.81 | 86.67 | 91.85 | 97.04 |
| LR_gaa_10 | 17.78 | 45.19 | 77.04 | 87.41 | 91.11 | 95.56 | 97.78 |
| MC_gaa_BSA | 5.93 | 22.22 | 57.78 | 68.15 | 80.74 | 88.89 | 95.56 |
| LR_gaa_BSA | 9.63 | 25.93 | 54.07 | 68.15 | 82.22 | 90.37 | 96.3 |
| MC_gaa_vdw | 8.15 | 29.63 | 63.7 | 79.26 | 89.63 | 96.3 | 99.26 |
| LR_gaa_vdw | 5.19 | 23.7 | 68.15 | 77.78 | 87.41 | 93.33 | 97.78 |
| MC_gaa_4-6_nat | 14.07 | 34.81 | 65.93 | 75.56 | 88.89 | 94.07 | 97.04 |
| LR_gaa_4-6_nat | 15.56 | 40.74 | 66.67 | 79.26 | 89.63 | 92.59 | 97.78 |
| MC_gaa_vdw_nat | 4.44 | 17.78 | 44.44 | 60.74 | 74.81 | 86.67 | 93.33 |
| LR_gaa_vdw_nat | 2.96 | 8.89 | 24.44 | 34.81 | 47.41 | 60.0 | 71.11 |

**Table I.** Percentage of 77 complexes in the test set for which at least one near-native solution can be found in the top 1, top 10, top 100, top 200, top 500, top 1000, and top 2000.

|  | top 1 | 10 | 100 | 200 | 500 | 1000 | 2000 |
| --- | --- | --- | --- | --- | --- | --- | --- |
| Random | 0.0 | 0.0 | 10.39 | 18.18 | 33.77 | 42.86 | 58.44 |
| Attract | 6.49 | 22.08 | 46.75 | 55.84 | 68.83 | 83.12 | 89.61 |
| Tobi | 5.19 | 16.88 | 50.65 | 54.55 | 72.73 | 79.22 | 88.31 |
| MC_gaa_10 | 12.99 | 31.17 | 61.04 | 68.83 | 77.92 | 83.12 | 89.61 |
| LR_gaa_10 | 12.99 | 28.57 | 59.74 | 66.23 | 76.62 | 84.42 | 92.21 |
| MC_gaa_BSA | 6.49 | 24.68 | 55.84 | 68.83 | 83.12 | 84.42 | 94.81 |
| LR_gaa_BSA | 3.9 | 24.68 | 61.04 | 76.62 | 87.01 | 89.61 | 93.51 |
| MC_gaa_vdw | 6.49 | 16.88 | 57.14 | 66.23 | 81.82 | 88.31 | 92.21 |
| LR_gaa_vdw | 3.9 | 23.38 | 62.34 | 70.13 | 80.52 | 90.91 | 96.1 |
| MC_gaa_4-6_nat | 12.99 | 29.87 | 64.94 | 74.03 | 84.42 | 85.71 | 92.21 |
| LR_gaa_4-6_nat | 5.19 | 22.08 | 67.53 | 72.73 | 88.31 | 89.61 | 97.4 |
| MC_gaa_vdw_nat | 3.9 | 9.09 | 27.27 | 36.36 | 50.65 | 58.44 | 66.23 |
| LR_gaa_vdw_nat | 1.3 | 3.9 | 29.87 | 40.26 | 58.44 | 80.52 | 90.91 |

**Table J.** Average fraction of all near-native solutions for 135 complexes in the training set in the top 0.1, 1, 2, 5, 10 and 20 % of all generated decoys by ATTRACT.

|  | 0.1% | 1% | 2% | 5% | 10% | 20% |
| --- | --- | --- | --- | --- | --- | --- |
| Random | 0.15 | 1.15 | 2.14 | 4.73 | 9.89 | 18.88 |
| Attract | 3.75 | 14.05 | 20.56 | 32.69 | 44.81 | 60.46 |
| Tobi | 1.94 | 7.49 | 10.99 | 18.03 | 26.75 | 40.87 |
| MC_gaa_10 | 7.6 | 27.15 | 37.79 | 55.49 | 71.06 | 84.76 |
| LR_gaa_10 | 9.65 | 31.64 | 42.87 | 59.85 | 73.16 | 84.76 |
| MC_gaa_BSA | 4.83 | 18.94 | 27.1 | 44.34 | 61.56 | 76.44 |
| LR_gaa_BSA | 4.37 | 17.62 | 26.69 | 43.32 | 59.52 | 76.61 |
| MC_gaa_vdw | 3.67 | 14.48 | 22.99 | 39.71 | 54.0 | 70.58 |
| LR_gaa_vdw | 4.13 | 17.99 | 26.26 | 41.38 | 54.49 | 72.5 |
| MC_gaa_4-6_nat | 5.07 | 15.76 | 23.25 | 36.87 | 48.64 | 65.74 |
| LR_gaa_4-6_nat | 5.56 | 19.43 | 27.32 | 42.3 | 56.6 | 72.79 |
| MC_gaa_vdw_nat | 1.66 | 6.74 | 9.8 | 17.46 | 28.47 | 45.92 |
| LR_gaa_vdw_na | 0.45 | 1.89 | 2.91 | 5.28 | 7.64 | 13.25 |

**Table K.** Average fraction of all near-native solutions for 77 complexes in the test set in the top 0.1, 1, 2, 5, 10 and 20 % of all generated decoys generated by ATTRACT.

|  | 0.1% | 1% | 2% | 5% | 10% | 20% |
| --- | --- | --- | --- | --- | --- | --- |
| Random | 0.28 | 1.2 | 1.83 | 4.11 | 8.4 | 19.43 |
| Attract | 4.89 | 16.63 | 26.56 | 38.93 | 52.63 | 66.66 |
| Tobi | 2.41 | 10.96 | 15.62 | 25.89 | 35.35 | 48.05 |
| MC_gaa_10 | 13.7 | 39.76 | 49.92 | 63.65 | 74.83 | 86.04 |
| LR_gaa_10 | 10.93 | 33.69 | 42.94 | 60.07 | 72.14 | 82.75 |
| MC_gaa_BSA | 7.13 | 33.7 | 44.96 | 60.21 | 72.94 | 85.85 |
| LR_gaa_BSA | 5.62 | 32.82 | 43.21 | 59.43 | 73.63 | 85.65 |
| MC_gaa_vdw | 4.67 | 20.81 | 31.13 | 47.82 | 62.26 | 77.26 |
| LR_gaa_vdw | 4.71 | 23.15 | 33.63 | 51.3 | 64.47 | 80.28 |
| MC_gaa_4-6_nat | 9.23 | 25.8 | 34.07 | 48.67 | 60.25 | 72.13 |
| LR_gaa_4-6_nat | 6.81 | 25.57 | 37.97 | 54.91 | 67.46 | 80.78 |
| MC_gaa_vdw_nat | 0.9 | 4.04 | 6.37 | 8.71 | 11.91 | 17.16 |
| LR_gaa_vdw_nat | 0.83 | 7.47 | 12.16 | 23.83 | 37.03 | 54.28 |
